# Frequency of factors that complicate the identification of mild traumatic brain injury in level I trauma center patients

**DOI:** 10.2217/cnc.15.11

**Published:** 2015-11-16

**Authors:** Robyn E Furger, Lindsay D Nelson, E Brooke Lerner, Michael A McCrea

**Affiliations:** 1 Department of Neurosurgery, Medical College of Wisconsin, Milwaukee, WI, USA; 2 Department of Neurology, Medical College of Wisconsin, Milwaukee, WI, USA; 3 Department of Emergency Medicine, Medical College of Wisconsin, Milwaukee, WI, USA

**Keywords:** assessment, comorbidities, concussion, confounding variables, emergency department

## Abstract

**Aim:**

Determine the frequency of factors that complicate identification of mild traumatic brain injury (mTBI) in emergency department patients.

**Setting:**

Chart review.

**Materials & methods:**

Records of 3042 patients (age 18–45 years) exposed to a potential mechanism of mTBI were reviewed for five common complicating factors and signs of mTBI.

**Results:**

Most patients (65.1%) had at least one complicating factor: given narcotics in the emergency department (43.7%), on psychotropic medication (18.4%), psychiatric diagnosis (15.3%), alcohol consumption near time of admission (14.2%) and preadmission narcotic prescription (8.9%).

**Conclusion:**

Our findings highlight the frequency of these confounding factors in this population. Future research should identify how these factors interact with performance on assessment measures to improve evidence-based mTBI assessment in this population.

Mild traumatic brain injury (mTBI) is a major public health problem. The CDC estimated that in 2010, approximately “2.5 million emergency department (ED) visits, hospitalizations, or deaths were associated with TBI” [1,2], the vast majority of which (at least 70–90%) can be classified as mild in nature [3]. Although the majority of individuals with mTBI recover spontaneously within several days or weeks [4,5], a substantial minority report persistent symptoms, occupational impairment and other disability [6,7]. Given increased recognition of this injury as highly prevalent and costly to individuals and society, there has been an influx of research on the clinical signs, underlying pathophysiology and natural course of recovery following mTBI.

Research on mTBI faces many challenges due to the variable biomechanics of head impact and injury severity across patients as well as the heterogeneity of the people who sustain and seek medical care for head injury [8–10]. As mTBI symptoms are highly nonspecific, a number of non-mTBI factors can present in concert with mTBI that complicate the assessment of the sequelae (e.g., symptoms, cognitive impairment) of head trauma. For example, premorbid psychiatric conditions, which are common in the general population [11], are associated with increased reporting of ‘postconcussive’ symptoms [12–14], and some of the events that cause mTBI in ED patients (e.g., assaults, motor vehicle crashes) may cause more severe stress reactions than sport-related concussions [15]. Furthermore, orthopedic injuries that are common in patients with TBI [16] cause similar symptoms and functional impairments as mTBI [17,18], and some medications and drugs (taken prior to admission or for treatment of pain acutely after injury) are likely to complicate identification/assessment of mTBI and recovery from injury [19–21]. Along with these challenges is also the initial challenge of simply diagnosing mTBI which has been historically unclear due to its nonspecific clinical symptoms, multiple definitions of the injury and limited methods of objectively identifying the injury [22,23].

Unfortunately, research samples that have included mTBI patients from broad hospital-based patient populations have made it difficult to tease apart to what degree findings were attributable to mTBI or these other factors. In fact, a 2004 review of the mTBI literature by the WHO Center for Neurotrauma Task Force on Mild Traumatic Brain Injury concluded that much of the existing research base was lacking even in descriptions of the samples’ preinjury characteristics and other potentially complicating factors [9]. The authors called for future research to focus on distinguishing between mTBI symptoms and ‘factors such as pain, medication effects, psychological distress and litigation/compensation’ such that apparent mTBI effects would not be unknowingly magnified by other variables [9].

In response to this problem, researchers have taken two major approaches to better isolate mTBI from other common comorbidities: recruit from populations without common complicating factors; and recruit from complex patient settings but apply stricter exclusionary criteria. The sport-related concussion literature has provided an invaluable model of the former approach by identifying healthy individuals at risk for sustaining concussion, recognizing injuries as they occur and facilitating selection of well-matched noninjured control samples (i.e., uninjured teammates) [24,25]. Applying the second approach, other researchers have recruited from typical hospital-based patient samples while excluding patients with psychiatric conditions and other complicating factors. This approach, however, has resulted in very limited samples of patients to study (i.e., <5% of the mTBI population in two Finnish samples) [8,26]. Although these research strategies are invaluable for evaluating the clinical and neurobiological effects of concussion in very healthy individuals, it is unknown to what degree findings from such research can be generalized back to the broader head injured population. While there has been an increase in higher quality concussion related research in recent years, gaps still remain in the operationalization and diagnosis of the injury as well as management during recovery [22,27].

Here, we present data from the efforts of our laboratory to recruit a relatively pure sample of mTBI patients from a level I trauma center ED in the Milwaukee, Wisconsin region. This study was part of a larger project funded by the Department of Defense (DoD) which required subjects to be free of several confounding variables so as to isolate the true effects of mTBI. As a complement to the Finnish data presented by Luoto and colleagues [8,26], we report on the frequency of the five confounding factors that were considered as possible complications prior to enrollment for the larger study. To our knowledge, this is the first set of data from a US population to reproduce similar struggles for mTBI research enrollment as Luoto and colleagues’ studies. The findings shed light on the relative frequency of these confounding factors in the hospital-based mTBI population and highlight the difficulties inherent in conducting meaningful patient-based mTBI research. Presentation of these data follows with a discussion of how future research efforts could more systematically tease apart the interactions among various complicating factors and mTBI in order to better understand the effects of the injury across differing types of patients.

## Materials & methods

### Participants

We conducted a prospective chart review of every patient treated in the ED at a tertiary care hospital that also serves as the area's level I trauma center in Milwaukee, Wisconsin during a 1-year period (August 2012 through July 2013). Inclusion criteria for chart review were being in the age range of interest (18–45 years) and having been exposed to one of the most common mechanisms of injury that could lead to mTBI, according to the CDC [1]. These included falls, assaults, struck by/against an object and motor vehicle traffic crashes (MVT). MVT included: MVT–occupant (patient inside a car), MVT–motorcycle, MVT–bicycle, MVT–pedestrian (patient struck by a vehicle) and MVT–other. Sport-related injuries would largely have fallen into the struck by/against category as this was operationalized as events in which a person was struck unintentionally by another person or an object. Patients were excluded if their Glasgow Coma Scale score was less than 13. The age range was selected to match the inclusion criteria of the larger study of mTBI in ED patients, on which this study was based. In total, 3042 patients met our inclusion criteria and were included the analyses below.

### Operationalization & extraction of variables of interest

These patients were identified as part of a larger research study approved by our local Institutional Review Board. Informed consent was not necessary as these data were part of screening procedures for enrollment in the larger research study. The complicating factors for assessing an mTBI patient as a possible research participant were selected by the study's advisory board based on their relevance to our targeted aims. These factors included presence of a current primary Axis I diagnosis (according to the Diagnostic and Statistical Manual of Mental Disorders – Fourth Edition [DSM-IV-TR]) [12–14,28], currently using prescribed psychotropic medications, currently using prescribed narcotics [19,21], having had narcotics administered in the ED [20] and indication of alcohol consumed around the time of injury [21]. Note that while a psychiatric history or home narcotic prescription were considered exclusionary criteria for involvement in the study, acute narcotic administration or recent alcohol intoxication were considered to be factors that could complicate the assessment of mTBI symptoms [29,30] and thereby would necessitate testing after the acute effects of these drugs had worn off. The study coordinator also coded mention of any signs (loss of consciousness, amnesia) or symptoms of mTBI documented in the patient charts, with potential symptoms extracted from the DoD definition of mTBI [31]. The most commonly observed signs and symptoms, however, were restricted to headache, loss of consciousness, nausea, dizziness/balance problems and amnesia.

Data were extracted from each patient's medical record by a full-time research coordinator using a structured electronic data form. At the onset of the study, the research coordinator and study PI examined the format and content of patient records and developed a protocol dictating from what fields to extract each variable of interest and how to operationalize the variables of interest. In particular, home psychotropic and narcotic prescription information was extracted from the medication history and outpatient medication lists. Narcotics that were administered in the ED were documented in a list of medications given within the ED visit. Axis I diagnoses were extracted from patients’ past medical history form. Alcohol was defined as any mention of alcohol in the nursing notes. Mechanism of injury was determined based on the patient's documented chief complaint or the healthcare provider's free text summary of the ED encounter. For cases in which information was ambiguous or unusual, a second rater independently coded the information, and if their two codes were discrepant, the raters discussed the case to achieve consensus. Such ambiguities were most common for the mechanism of injury variable. The individual variables that make up the DoD definition of concussion were also extracted: specifically, whether or not any specific acute injury characteristic (i.e., loss of consciousness, amnesia, altered mental status) *or* symptom (see list above) of mTBI was present [31]. These factors were identified by reviewing the physician and nurses’ free text notes.

### Data analysis

As the aim of the study was mostly descriptive, data analyses consisted primarily of descriptive statistics. Frequency statistics including percentages were calculated. Chi square was also used to compare patients with versus without documented mTBI signs and symptoms for demographic variables (age, sex), mechanism of injury and rates of complicating factors. Statistical analyses were conducted in R (v. 3.0.3) [32].

## Results

### Sample characteristics

The distribution of patients by mechanism of injury was: motor vehicle-traffic crashes (62.4%), falls (21.3%), assaults (13.1%) and struck by/against (3.2%). Motor vehicle-traffic crashes were distributed by subcategory as follows: occupant (85.6%), motorcycle crash (7.5%), pedestrian struck (4.6%), bicycle struck (1.9%) and other (0.4%). Patients were roughly half male (52.0%) and averaged 30.0 years old (standard deviation: 7.8).

### Frequency of complicating factors

**Figure 1.**
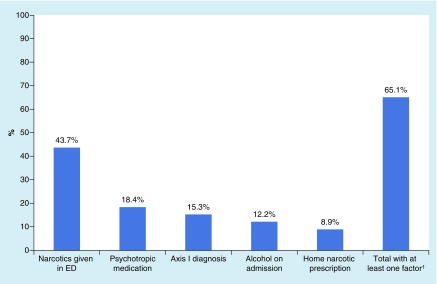
**Frequency of complicating factors in patients presenting with a common mechanism of mild traumatic brain injury (n = 3042).** ^†^Equals aggregate total percentage of sample with at least one of the five factors listed. ED: Emergency department.

Figure 1 shows the frequency of each factor. Overall, 65.1% of the sample had at least one targeted complicating factor (16.8% had two, 7.5% had three, 1.2% had four and none had all five).

### Comparisons of patients with & without mTBI symptoms

A total of 882 patients (29.0% of the sample) had documented acute injury characteristics and/or symptoms of mTBI. Table 1 illustrates that the demographics were similar between the groups with and without documented symptoms. Group differences in age, although statistically significant (p = 0.002), were likely not clinically significant (d = 0.13). Certain mechanisms of injury were more likely to be associated with the presence of mTBI symptoms, χ^2^(3) = 80.03; p < 0.001, with struck by/against events most likely to be associated with documented mTBI symptoms (58.2%), followed by assaults (40.9%), motor vehicle-traffic (26.6%) and falls (24.3%). *Post hoc* pairwise tests (adjusted for multiple comparisons using the false discovery rate control method [33]) were all significant (p < 0.01) with the exception of the fall versus motor vehicle-traffic categories (p = 0.241).

**Table 1.** **Comparisons of patients with versus without documented mild traumatic brain injury symptoms on demographics, mechanism of injury and presence of complicating factors (n = 3042).**

	**Symptomatic (n = 882)**	**Asymptomatic (n = 2160)**	**p-value**
Gender (male)	51.5%	52.3%	0.691

Age, mean (standard deviation); years	29.3 (7.5)	30.3 (7.9)	0.002

**Complicating factor**			

Narcotics given in the emergency department	42.0%	44.4%	0.226

Psychotropic medication	20.1%	17.8%	0.139

Axis I diagnosis	16.9%	14.6%	0.115

Alcohol on admission	17.5%	12.8%	0.001

Home narcotic prescription	10.3%	8.3%	0.081

Rates of complicating factors between the mTBI symptomatic and asymptomatic groups were statistically different only for alcohol, with symptomatic patients more likely to have consumed alcohol around the time of admission (17.5 vs 12.8% in those without mTBI symptoms). There were no differences between the symptomatic and asymptomatic groups on the frequency of other complicating factors.

## Discussion

In this large sample (n = 3042) of ED patients who presented to a level I trauma center with a common mechanism of injury leading to mTBI, we found that a large majority (65%) have at least one co-occurring factor that could complicate the identification/assessment of mTBI and potentially their recovery from injury. These results, alongside parallel findings from two Finnish samples [8,26], highlight the circular problem whereby efforts to recruit ‘clean’ samples of mTBI patients (those with mTBI and no relevant comorbidities) may greatly limit study enrollment, and in turn study cohorts are not at all representative of the actual population of individuals affected by mTBI. This approach may inherently limit generalizability of findings to the true population and therefore to everyday clinical practice. The underlying issue is that in studying mTBI only in ‘clean’ samples, our ability to understand interactions between comorbid factors, presentation of acute head injury and postinjury recovery is limited. For example, previous research has shown that individuals with preexisting mental health conditions are at increased risk for poor outcome and postconcussive symptoms [34,35]. Further, our data suggest that patients presenting with recent alcohol consumption presented more frequently with documented signs and symptoms of mTBI, suggesting either that alcohol artificially elevates such symptoms or that this group has a higher prevalence of mTBI. The larger research study from which this project was derived applied exclusionary criteria related to preexisting mental health conditions and other factors with the aim of isolating the effects of mTBI. However, this practice inherently limits our ability to generalize the findings to the modal mTBI patient for whom complicating factors present alongside head trauma. An important question for these patients is to what degree various comorbid factors present with overlapping versus distinct features.

Furthermore, it is unknown to what degree the effects of different complicating factors result in additive or interactive effects with mTBI. There remains a great need to develop techniques and standards for diagnosing mTBI in emergency or urgent care settings and, outside the ED setting, in monitoring recovery from the injury. As such, future research in civilian, nonathlete mTBI patients will need to more systematically tease apart the influence of differing potentially complicating factors in symptom presentation or performance on standardized assessment tools being studied in the population. Accumulation of large samples will be necessary such that the clinical sequelae of differing individual confounding factors and combinations of factors can be studied with reference to features of clinical presentation and degree of impairment on varying types of exams (e.g., symptoms, neurocognitive tasks, postural instability and biomarker assessment).

For example, it would be valuable to explore the degree to which assessment tools validated for sport-related concussion, developed on individuals relatively free of these complicating factors [36], generalize to the ED setting. This is important because mTBI is a prevalent and costly injury for which formal assessment and treatment tools have not been well validated for civilian nonathlete patients (e.g., ED patients), despite the fact that the majority of patients with mTBI fall into this category [37]. These findings support the idea that a major reason why it may be difficult to develop and validate diagnostic and clinical management procedures for these patients is that they represent a heterogeneous population who frequently present with comorbid/confounding factors that cause similar signs/symptoms as mTBI. Given the high prevalence of complicating factors present in this population, findings from studies employing such exclusionary criteria may not have ecological validity for the typical mTBI patient. To the extent that research programs can recruit and follow a wide variety of mTBI patients (e.g., those varying in demographic characteristics and presence of complicating factors), the relative importance of various confounding variables in mTBI assessment can be identified. These factors may also carry prognostic value for recovery from mTBI, further supporting inclusion of patients with and without such variables in studies aimed at predicting recovery from injury. By clarifying the specific variables that do and do not confound mTBI assessment and recovery, research programs will be better able to capitalize on their available patient populations without sacrificing the integrity of their findings.

The diagnostic utility of common concussion assessment tools for identifying the subjective (symptoms) and objective (changes on performance-based measures of cognition, balance) indicators of head injury is significantly limited without a more complete understanding of how complicating factors affect symptom reporting and test performance. In contrast to the sports medicine setting, identifying how these factors will influence ED testing for mTBI is essential because ED clinicians working in a trauma setting will rarely have the benefit of knowing patients prior to their arrival in the ED and will rarely have baseline cognitive and symptom data. By building normative datasets on patients more similar to those encountered in ED settings, appropriate clinical decision rules (e.g., normative cutoff scores) could be identified for the types of patients who are likely to present with mTBI in EDs. Ongoing national initiatives to aggregate data across samples and settings (e.g., Transforming Research and Clinical Knowledge in TBI [TRACK-TBI], TBI Endpoints Development [TED] project, Chronic Effects of Neurotrauma Consortium [CENC] [38–40]) are well poised to contribute such data. Targeted studies aimed at recruiting target populations that vary on one or more confounding factors may also be necessary to ensure findings of relevance to the varying clinical populations who are treated for mTBI.

The complicating factors recorded in the current study were selected due to their suspected prevalence in the ED population and confounding influence on mTBI identification and research. There are numerous ways in which preexisting psychiatric conditions, medications and drugs of abuse could be expected to confound identification, assessment and research, particularly with respect to mental status and cognitive assessment. It will be valuable for future studies to elucidate the relative influence of these factors on specific aspects of mTBI assessment (which commonly target multiple areas of functioning, including self-reported symptoms, cognitive performance and postural stability). As symptom checklists require ratings of emotional, somatic and cognitive symptoms [41,42], it is possible that premorbid psychiatric conditions (e.g., depression, which involves low mood as well as physical and cognitive symptoms like fatigue and difficulty concentrating) could elevate baseline ratings on such checklists. Furthermore, acute intoxication due to alcohol or narcotic pain analgesia delivered in the ED could alter symptom ratings. Certainly, alcohol or drug related intoxication would also be expected to temporarily impair performance on measures of cognitive performance [36] or postural stability (balance) [4,43], both of which are frequently used in the assessment of sport-related concussion [44]. Further, individuals presenting to the ED with a history of alcohol or other drug (e.g., cocaine) abuse are probably more likely than those who do not abuse substances to manifest premorbid cognitive impairment [45]. The extent to which narcotic medications would affect performance on objective measures of cognitive abilities or balance is somewhat unclear but might be more marked in individuals without a history of taking such medications who receive it for the treatment of acute pain [20]. In contrast, individuals using narcotics preadmission may have a tolerance to these drugs and, therefore, may not show noticeable effects in performance [46].

Given the method of data collection in this study (i.e., review of medical records), it is possible that some variables (e.g., axis I condition, presence of alcohol) were not consistently documented [37,47], and consequently our findings probably represent lower bounds for the true frequency of these complicating factors. Additionally, because of the manner in which we collected data, there are likely variables of interest that were not available (e.g., race, socioeconomic status, education level, CT scans). Future work might include a broader age range and capture more nuanced data about patients’ premorbid histories and outcomes in order to explore how rates of these co-occurring factors vary by other factors. As our inclusion criteria were broad given our aims, it is possible that the demographics of our sample do not match those of the broader mTBI population. For example, that our sample of possible mTBI patients was roughly half female is somewhat surprising in light of work suggesting that males are at greater risk of sustaining mTBIs [3]. However, because this variable was not predictive of the complicating factors targeted in this report, such sampling issues are not expected to affect our core findings (frequency of these complicating factors in the ED population).

In summary, this study represents an important step toward gaining a better understanding of how co-occurring factors may complicate the identification, assessment and research of mTBI. Future work should identify how these factors interact with performance on assessment measures and postinjury recovery so that clinicians will be better able to assess for mTBI in this complex and heterogeneous population. Ultimately, this line of research is aimed at improving diagnostic accuracy, directing acute triage and informing resource utilization for patients affected by mTBI, all in an effort to facilitate recovery and improve outcome after mTBI.

**Executive summary**

- Research on mild traumatic brain injury (mTBI) is challenged by the nonspecific nature of the symptoms used clinically to diagnose the injury and the numerous factors that may co-occur in patients with mTBI that cause similar clinical features.
- We conducted a 1-year, prospective chart review of over 3000 patients presenting to the emergency department at a level I trauma center with common mechanisms of mTBI in order to estimate the prevalence of several complicating factors of interest.
- A large majority of patients (65.1%) presented with at least one documented factor that would complicate the assessment of mTBI (e.g., having been given narcotics in the emergency department, having evidence of preinjury psychiatric issues, being acutely intoxicated with alcohol).
- Considering these findings, future research on civilian mTBI should aggregate large, heterogeneous samples of patients with these complicating factors to enable researchers to systematically tease apart the pattern and magnitude of effect of mTBI versus these other factors on presentation of acute head injury and postinjury recovery, all in an effort to improve our ability to recognize and treat mTBI in the broader patient population.
